# Urban form and productivity: What shapes are Latin-American cities?

**DOI:** 10.1177/2399808321999309

**Published:** 2021-03-08

**Authors:** Juan C Duque, Nancy Lozano-Gracia, Jorge E Patino, Paula Restrepo

**Affiliations:** 28008 Universidad EAFIT, Colombia; World Bank, Sustainable Development Department -- MENA; 28008 Universidad EAFIT, Colombia; World Bank, Urban, Resilience and Land Global Practice -- LAC

**Keywords:** Productivity, urban form, nighttime lights, Latin America

## Abstract

This paper examines the linkages between urban form and city productivity using seven alternative metrics for urban form and applying them to a comprehensive sample of Latin-American cities. While most of the literature has concentrated on the effects of population density (compact vs. sprawling urban development), this paper seeks to assess whether different dimensions of a city’s urban form, such as shape, structure, and land use, affect its economic performance. We found that both the shape of the urban extent and the inner-city connectedness have a statistically significant association with the productivity level of a city.

## Introduction

Early work on urban economics recognized the links between urban form and economic performance (Parr, 1979). The spatial structure of cities is thought to have an important influence on the emergence of agglomeration economies and congestion costs and, hence, on a city’s level of productivity, sustainability, and quality of life (Squires, 2002). The same channels—matching, learning and sharing—used to explain the emergence of agglomeration economies (Duranton and Puga, 2004) are also the links between urban form and city productivity. Local governments, through land use regulations and other urban policy instruments, can influence the locations of economic activities, urban infrastructure, and households (World Bank, 2009). These differences in the occupation and density of urban spaces have consequences in terms of the transport modes used, commuting times, and type and intensity of human interaction (Cervero, 2001; Ciccone and Hall, 1996; Rosenthal and Strange, 2004). However, despite these links being well established through a theoretical framework, there are few empirical studies that examine the relationship between urban form and economic performance, and those that do mainly focus on developed economies.

With over 80% of Latin America and the Caribbean’s (LAC) population living in cities (UN, 2018), understanding the links between city form and productivity is of paramount importance for policy makers in the region. The region’s challenging topography, along with the rapid urbanization it has undergone with limited infrastructure investments, may have led to urban forms that pose a barrier to the rise of agglomeration economies, limited firm interaction, and increased spatial mismatch. A better understanding of the links between urban form and productivity can shed light on whether urban policy has a role to play in supporting city productivity and, through such productivity, economic growth.

In this paper, we examine the linkages between urban form and city productivity using seven urban form metrics for a comprehensive sample of Latin-American cities. We use a consistent time series of Defense Meteorological Satellite Program – Operational Linescan System (DMSP-OLS) nighttime lights (NTL) imagery to identify city extents, characterize urban form and create a proxy measure of a city’s economic performance. In terms of the methodological approach, our first base model follows Fallah et al. (2011) and includes the estimation of urban productivity using time-lagged measures of urban form and other control variables. In a second model, we follow Harari (2016) and implement a synthetic instrument that uses the potential shape of a city to calculate the form indicators based on such potential shape. The contribution of this paper is twofold. First, by seeing urban form as a concept that goes beyond population density, we show that land use, transport, and other city planning policies are important instruments that local governments have, to foster productivity in cities. Second, we use a methodological approach based on open data that are available on a global scale and open source tools, which allows us not only to provide empirical evidence for more than 900 Latin-American cities but also to easily replicate the method in other cities across the globe.

The rest of the paper is organized as follows. The following section provides a literature review. Next sections present the empirical models and describe the source data and the construction of the proxy measure of a city’s economic performance, the measures of urban form, and the construction of the synthetic instrument. Then the empirical results are presented and, finally, our conclusions are presented.

## Literature review

Population density and city size have been commonly referenced in the economic literature as key aspects of urban productivity. The most common conclusion is that less dense cities face higher commuting rates (Wheeler, 2001), higher marginal costs of transporting intermediate goods (Ciccone and Hall, 1996), and lower knowledge spillovers (Glaeser, 1998; Jaffe et al., 1993; Lynch, 1981). Conversely, other authors state that the productivity costs of sprawling are being reduced by improved transportation networks, public transport systems (Chatman and Noland, 2014; Glaeser and Kahn, 2004) and advances in information and communications technologies (Partridge et al., 2008).

However, although simple to calculate, neither density nor size can capture the multidimensional nature of urban form (Cutsinger et al., 2005; Galster et al., 2001). These aggregated variables assume a uniform distribution of people across space and do not capture the variations in urban structure and land use (Lopez and Hynes 2003; Melo et al., 2017). Currently, thanks to the evolution of geographic information science and remote sensing, together with an increase in the availability of georeferenced data, urban economists are looking beyond density and size when exploring the relationship between urban form and city productivity. An example of this trend can be found in Tewari et al. (2016), who, in Indian cities, find a positive and statistically significant impact of a city’s initial level of geometric compactness on its subsequent economic growth (estimated on the basis of NTL data). Fallah et al. (2011), who explore the relationship between urban sprawl and labor productivity, use information at the census block level to develop a sprawl measure that captures differences in the distribution of population density within the city.

Urban planners, in the search for an integrated theory of city planning, have explored multidisciplinary approaches that incorporate areas such as economics, network science, and geometry to understand the impact of size, scale, and shape on city sustainability (Batty, 2008). This process has led them to have a more elaborate conception of urban form. Contributions such as those of Batty and Longley (1994), Prosperi et al., (2009), and Whyte (1968) conclude that a proper characterization of a city’s form should include information on the shape of its border, its urban texture and land use planning. Based on these characteristics, urban planners differentiate between natural/organic and planned/regular/artificial/geometric cities. Planned cities are characterized by straight streets, circular borders, and a clear segregation of land uses. Organic cities tend to have open spaces that are randomly located, curved roads, and borders that follow the natural landscape more closely.

Our contribution to the literature consists of using the multidimensional urban planning definition of urban form to attain an integral understanding of the association between urban form and city productivity. We also contribute to the literature by providing empirical evidence for developing countries, and for the first time, providing a comprehensive analysis of urban form and productivity in a large sample of LAC cities.

## Model

To examine the relationship between urban form and productivity, we adopt the following specification in equation (1)

(1) $$Y_{\mathrm{it}}=\alpha S_{\mathrm{it}}+\beta T_{\mathrm{it}}+\gamma L_{\mathrm{it}}+\xi X_{\mathrm{it}}+\theta_{i}+\epsilon_{i},$$

where $Y_{it}$ is the productivity of city *i* at year *t*; S is a vector of urban shape variables; T is a vector of urban texture variables; L is a vector of land use pattern variables; and X is a vector of control variables, including the intercept. θ includes the country fixed effects, and ϵ is the error term.

This formulation requires dealing with endogeneity issues in the relation between urban form and productivity: on the one hand, urban form affects productivity through the interaction between congestion costs and agglomeration economies. On the other hand, local governments in productive cities are more likely to invest resources in planning the city’s urban form. For identification purposes, this work explores two alternative strategies:

*Strategy 1:* Following Fallah et al. (2011), we use lagged explanatory variables to mitigate possible direct simultaneity between urban form and city productivity (see equation (2)). We expect that the channels through which city form affects productivity are not immediate and may take time. Therefore, considering data availability, we use a 10-years lag (i.e. *k *=* *10; Fallah et al. 2011, use a lag of 11 years). We recognize that, although commonly used, lagged explanatory variables are not the most effective way to address endogeneity. Thus, the results in this strategy are better interpreted as correlations.

(2) $$Y_{\mathrm{it}}=\alpha S_{i,(t-k)}+\beta T_{i,(t-k)}+\gamma L_{i,(t-k)}+\xi X_{i,(t-k)}+\theta_{i}+\epsilon_{i}$$

*Strategy 2:* Following Harari (2016), we instrument the actual shape (urban footprint) of each city-year with its potential shape, which results from a concentric expansion path. The estimation has the following form (see equations (3) and (4))

(3) $$Y_{\mathrm{it}}=\alpha S_{\mathrm{it}}+\delta X_{\mathrm{it}}+\theta_{i}+v_{t}+\epsilon_{\mathrm{it}}$$

(4) $$S_{\mathrm{it}}=\sigma \hat{S_{\mathrm{it}}}+\zeta X_{\mathrm{it}}+\omega_{i}+\varphi_{t}+\pi_{\mathrm{it}}$$

where $\hat{S}$ is a vector of city shape indicators derived from the potential urban extent; θ and  ω are the country fixed effects; v and φ are the year fixed effects; finally ϵ and π are the error terms.

For completeness, we present the steps proposed by Harari (2016) to estimate the potential urban extent, assuming a common average expansion rate across all cities:

1. For the first year, the potential urban extent is the largest patch of developable land (i.e. excluding water bodies and steep terrains) within the minimum-bounding circle enclosing the real urban extent of that first year.

2. Estimate the predicted area of city *i* in year *t*, $\hat{{\mathrm{area}}_{i,t}}$ , with equation (5)

(5) $$\log\left({\mathrm{area}}_{i,t}\right)=\theta_{i}+\gamma_{t}+\epsilon_{i,t}$$

  where $\theta_{i}$ and $\gamma_{t}$ are country and year fixed effects. To estimate this regression, it is necessary to have the actual areas of the urban extents of all the studied cities for at least two different years.

3. The estimated urban extent of city *i* in year *t* consists of the largest patch of developable land within the circle of ratio $\hat{r_{i,t}}$ calculated with equation (6)

(6) $$\hat{r_{i,t}}=\sqrt{\frac{\hat{{\mathrm{area}}_{i,t}}}{\pi }}$$

## Data

### Urban extent delineation

To outline urban areas, we use the radiance-calibrated (RC) DMSP-OLS NTL data for 1996, 2000, and 2010 obtained from the NOAA National Centers for Environmental Information. We applied the deblurring process devised by Abrahams et al. (2018), which withdraws the light from the surroundings back to their source pixels within the city. We also performed the empirical intercalibration proposed by Hsu et al. (2015) to enable the comparison between years. Finally, we applied the interannual series correction proposed by Cao et al. (2016) to ensure that the lit pixels detected in an image do not disappear at a later date and that the lit pixel digital number (DN) values for each date are not smaller than the pixel DN value at the same location on a previous date (for technical details see Duque et al. (2019)).

**Figure 1.**
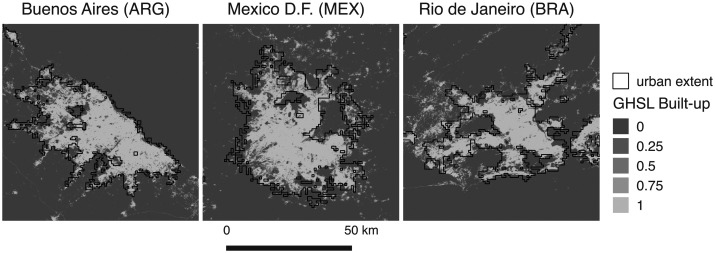
Examples of urban extents extracted from the 2000 NTL image over the built-up GHS reference layer for 2000.

We use the interannual corrected deblurred DMSP-OLS NTL RC images to outline urban extents in LAC. We applied a DN threshold to define what is considered an urban area in the NTL imagery (Harari, 2016; Li and Zhou, 2017; Zhang and Seto, 2011). We selected the data from the year 2000 and a sample of cities to check the pixel values where we could observe the transition from rural areas to urban areas. And because the nighttime images were previously empirically intercalibrated to enable the comparison between years, we applied the same threshold to the 1996 and 2010 images. As an additional check, the extents were compared to the built-up Global Human Settlements Layer (GHS) for the same year at 250 m of spatial resolution, GHS_BUILT_LDS1990_GLOBE_R2016A_ 54009_250 (Freire and Pesaresi, 2015). Figure 1 shows examples of the extracted urban extent and the built-up layer used as reference. Although the spatial resolution of the nighttime imagery used to extract the urban extents is four times coarser than the GHS built-up dataset used as reference (1 km vs. 0.25 km of pixel size), the examples in Figure 1 show that the obtained urban extents captured quite well those areas with more than 0.5 of built-up area in the GHS reference dataset.

**Figure 2.**
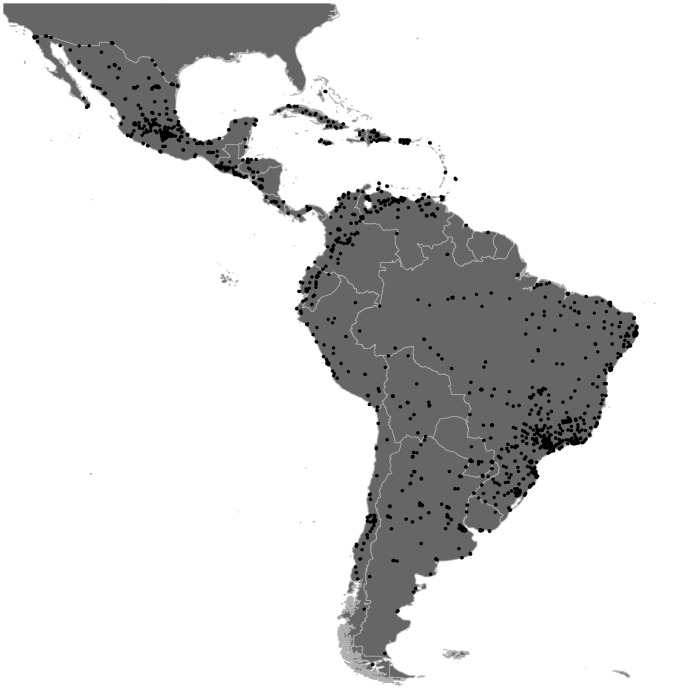
Urban areas in Latin America and the Caribbean extracted from the DSMP-OLS NTL 2010 image.

After we applied the threshold, we obtained binary images of the urban footprints in LAC cities. Those footprints were converted to vector format to create the polygons that outline the extent of cities. Finally, we applied a buffer of 10 m to the outlined polygons to merge all the polygons that belong to the same urban extent. By applying the 10-m buffer, we ensured that those pixels that were touching each other in a corner were part of the same polygon in the geospatial dataset. Although the buffer size is a fraction (0,01) of the pixel size of the NTL imagery, the impact on the quality of the urban extents is evident: it ensures the desired results without significantly affecting the shape of the obtained polygons. According to Chuvieco (2016), an object must be several times larger than the pixel size to be delineated properly from a remote sensing image. The pixel size of the DMSP-OLS NTL RC images is 30 arc seconds, which is almost 1 km × 1 km near the equator. As we were going to analyze the form of the urban extent, we excluded those urban extents with smaller sizes than 3 km^2^ to have better estimations of the urban form metrics (so the smallest urban extent is more than three pixels in the equator). Otherwise, we would have many very small urban extents that are all squared because they had only one or two pixels in the nighttime light imagery. The resulting final sample therefore includes 919 urban extents in each year (Figure 2).

### Measuring urban productivity

The use of NTL in socioeconomic studies is a response to the lack of economic measures at disaggregated scales. The pioneering contribution by Henderson et al. (2012) shows that NTL data can be used as a proxy of GDP within and across countries. Since then, other authors have used light density from NTL imagery to measure economic performance and welfare (Bleakley and Lin, 2012; Lowe, 2014; Michalopoulos and Papaioannou, 2013; Pinkovskiy, 2013; Storeygard, 2016). In this paper, we follow Tewari et al. (2016) to calculate a measure of productivity from NTL data. We use as our measure of productivity the density of radiance within the urban extent, dr_ntl_2010, computed as the sum NTL DN values in 2010 divided by the area, in square kilometers, of the urban extent in 2010.

**Figure 3.**
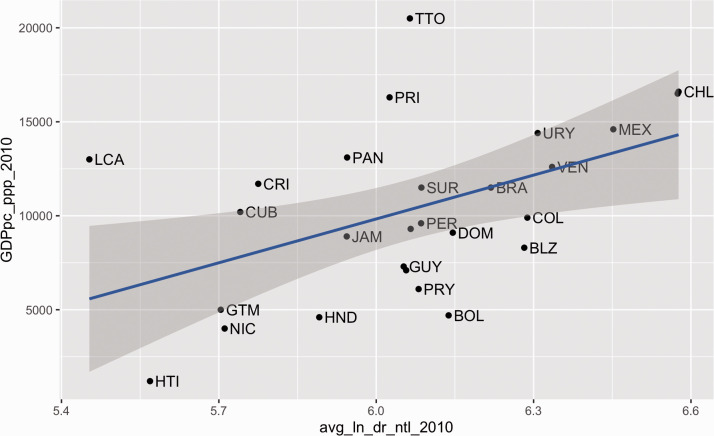
Country average ln(dr_ntl_2010) vs. GDPpc_ppp_2010. Note: Because of data availability, this plot does not include the following countries: Aruba, Barbados, Curaçao, Sint Maarten, and The Bahamas.

To show the potential of our productivity measure based on NTL imagery, we present in Figure 3 the relationship between our dependent variable in 2010 (ln_dr_ntl_2010) and the GDPpc_ppp_2010 obtained from the statistics database NationMaster (Holder, 2005). For this exercise, we had to change our scale from city to country because the GDP is available at the country level only. For this, we calculated the average of ln_dr_ntl_2010 between the cities within each country. The plot shows the expected direct relationship between both measures with a Pearson correlation coefficient of 0.48.

### Measuring urban form

From the literature on urban planning, we adopt an integral definition of urban form that includes three dimensions: shape of the urban extent, internal urban structure, and land use pattern. In this subsection, we present the metrics proposed for each of these dimensions, the rationale about the potential mechanisms through which each variable is supposed to influence economic performance, and the hypothesis to be tested in the empirical results.

A perfect circle has geometric properties such as minimum surface area and maximum accessibility from and to any interior point (Thompson, 1952). Angel et al. (2010) translate these geometric concepts into city shape and argue that the shape of a city affects its efficiency, equitability and sustainability. It is also proven that a city’s annual costs per household of public infrastructure and services are lower for circular/compact cities compared to fragmented/irregular/sprawling cities (Organization for Economic Co‐operation and Development, 2012). Thus, circular cities can reach efficiency at lower cost.

From a geometric perspective, a shape metric is usually focused on one of the two following geometric characteristics: the degree of roundness and smoothness of its perimeter (Angel et al., 2010). We used the ArcGIS tool Shape Metrics (Parent, 2011) to calculate two shape metrics: the exchange index, henceforth termed *roundness*, which measures how much the urban extent has deviated from its compact shape towards irregular noncompact forms, and the perimeter index, henceforth termed *smoothness*, which measures how smooth the perimeter of the urban extent is. Both metrics take the value one for a perfect circle.

Following the abovementioned rationale, our hypothesis regarding the association of these indicators of city shape with productivity is as follows:

**• H1**: An increase of the *roundness* or *smoothness* suggests a more circular urban extent, which is expected to be associated with higher productivity levels of a city.

As mentioned in the “Literature review” section, the internal structure of the city may play a considerable role in its productivity levels. Natural/organic cities have different dynamics than planned/regular/artificial/geometric cities. Such structures affect the way in which people and products move within the city. Mills and Hamilton (1989) and Bogart (1998) found that better accessibility to labor force and efficient transport infrastructure reduce time and costs, which increases productivity levels; Bertaud (2004) shows that shorter—and therefore cheaper—internal trips increase the levels of urban efficiency.

To capture such characteristics of the city structure, we used the OSMnx Python library (Boeing, 2017) to compute three geometry-based network topology variables: *Circuity_avg*, *Intersection_density*, and *Street_density*. Barrington-Leigh and Millard-Ball (2017) stated that globally, OpenStreetMap (OSM) data is approximately 83% complete by 2017 and this is improving with time. They also stated that “in many places, researchers and policymakers can rely on the completeness of OSM, or will soon be able to do so” (p. 14). We decided to use OSM data as it could be freely accessed and processed in an automated way using the Python OSMnx library. We are aware of the limitations of using that source of information, but it is the best information we could use for the entire Latin-American and Caribbean region. As the measures that we calculate from OSM data are global measures for each urban extent, we think that OSM data capture the cities’ general street network pattern quite well, even if it has some gaps or if it lacks some information in specific places.

The *Circuity_avg* measures the spatial inefficiency of a street network in connecting two points. *Circuity_avg* is calculated as the average ratio between the length of a segment and the straight-line distance between the two nodes it links (Boeing, 2017). Circuity values close to one indicate that the urban structure is dominated by regular street networks, and higher values indicate the presence of organic streets. According to Boeing (2018), the circuity values in the 27,000 US urban areas range between 1.02 and 1.14. As shown in Giacomin and Levinson (2015), for the most populated metropolitan statistical areas in the United States, low circuity is associated with more efficient and shorter trips. Using metropolitan areas in the United States, Huang and Levinson (2015) show that transit circuity affects the accessibility of transit networks. *Intersection_density* and *Street_density* give information about the ease of movement across the city (Boeing, 2017). Intersection density is calculated as the number of nodes divided by the area of the urban extent, considering only the set of nodes with more than one street connected to them, thus including only street intersections and excluding dead ends (Boeing, 2017). In the US urban areas, intersection density ranges between 12.47 and 49.42. Street density is calculated as the sum of the length of all segments of the street network (in this case measured in meters) divided by the area of the urban extent in km^2^ (Boeing, 2017). It ranges in the US urban areas between 4217 and 11,797.

From the discussion above, we formulate the following hypothesis related to the structure of the urban extent:

**• H2**: Regular urban structures (associated with low values of *Circuity_avg*) are usually associated with more efficient and shorter trips, which reduce agglomeration costs and may lead to higher levels of productivity.

**• H3**: An increase of either *Intersection_density* or *Street_density* implies higher levels of street coverage that facilitate the mobility of people and products, increasing the connectivity levels within the city, which is associated with higher productivity levels.

As shown in Fallah et al. (2011), the consideration of the distributional aspects of the population within the urban extent provides information on the land use pattern of the city. Fallah et al. (2011) proposed a measure of urban sprawl that allows differentiating between cities with even distributions of population from cities with highly concentrated populations. For completeness, we present in equation (7) the measure proposed by Fallah et al. (2011) and adopted in this work

(7) Sprawl=((L% − H%) +1) * 0.5

Dividing the urban extent into small areas, L% (H%) is the share of the urban population living in a small area with a density below (above) the median density calculated for the entire set of analyzed urban extents. We considered each pixel of 250 × 250 m from the GHS population layer to be a small area. Sprawl ranged from 0 to 1, with 1 indicating a greater level of sprawl. Fallah et al. (2011) find that higher levels of sprawl are associated with lower levels of productivity.

Finally, to measure *fullness*, the fraction of the urban extent that is built-up, we used the 1990 GHS built-up raster layer at a resolution of 250 m (Pesaresi et al., 2016). The GHS built-up layer values are expressed as decimals from 0 to 1 and correspond to the fraction of the pixel that is covered by a building. Fullness was measured as the mean value of all the pixels of the 1990 GHS built-up layer within the urban extent. Based on the extensive literature on the relationship between compact cities and productivity, we would expect a “full city” to be one that is also more compact, hence allowing for greater interaction and therefore higher agglomeration economies that ultimately increase productivity. However, a city that is “too full” might also suggest lack of public space, which can both be a disamenity and reflect a lack of planning. For these reasons, we can expect a nonlinear relationship between *fullness* and productivity.

**• H4**: The distribution of population density within a city affects its economic performance. According to Fallah et al. (2011), an uneven distribution of population density, associated with high levels of *sprawl*, can be linked to deteriorating socioeconomic outcomes, inefficient provision of public goods, and lower productivity levels.

**• H5**: An increase of the *Fullness_index* implies a less fragmented urban layout that may be associated with high productivity levels of the city, but, extremely high levels of fullness, can be correlated with decreasing productivity.

**Figure 4.**
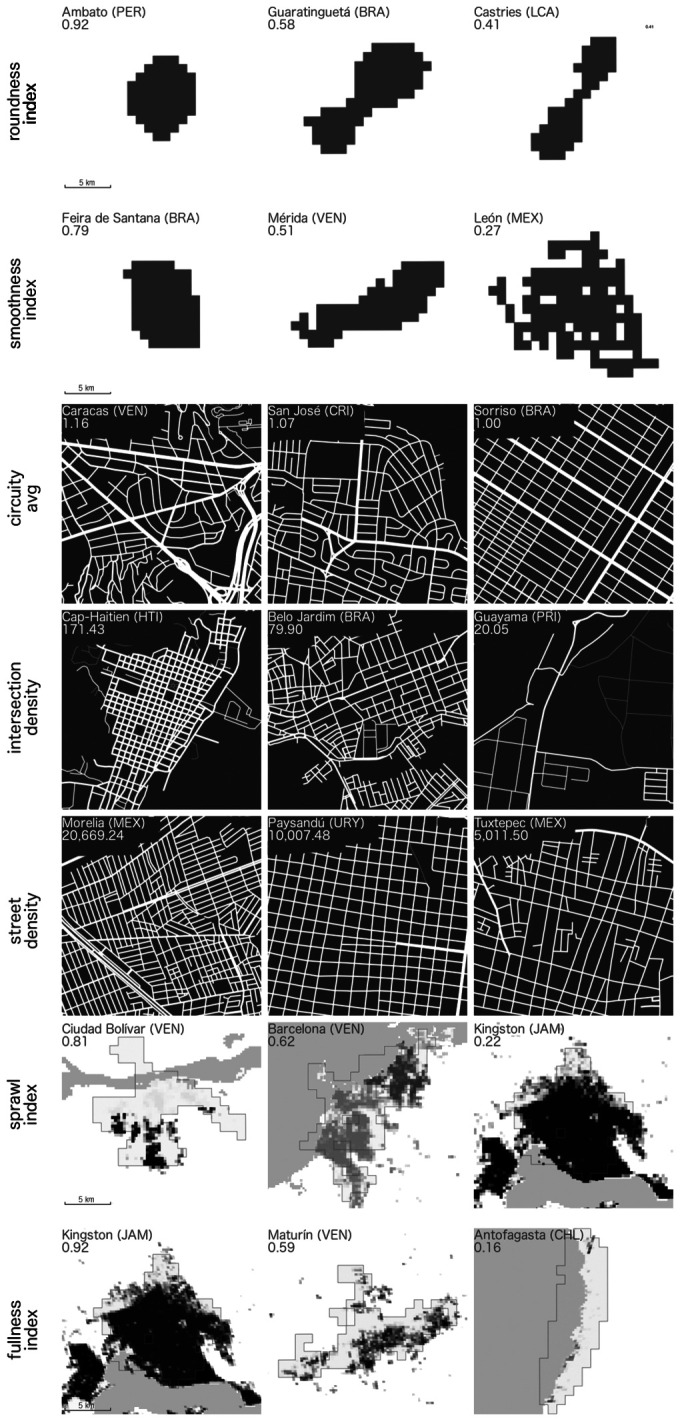
Examples of urban areas with high, medium and low values of the indexes that describe urban form.

In summary, Table 1 presents the metrics, their description, and data source for calculation, and Figure 4 presents examples of urban areas with high, medium and low values of each variable.

**Table 1.** Variables for describing urban form (vectors S, T, and L).

Dimension	Metric	Variable	Description	Data source for calculation
I. Urban Shape (*S*)	Roundness	Roundness_1996	Normalized share of the urban extent that is inside the equal-area circle around its center of gravity (Angel et al., 2010)	Urban extents from deblurred and corrected DMSP-OLS NTL RC 1996 image
	Smoothness of perimeter	Smoothness_1996	Normalized ratio between the perimeter of the equal-area circle and the perimeter of the urban extent (Angel et al., 2010)	Urban extents from deblurred and corrected DMSP-OLS NTL RC 1996 image
II. Urban Structure (*T*)	Urban structure	Circuity_avg_1996	Average ratio between the street-network distance and the straight-line distance between two points within the urban extent (Boeing, 2017)	OpenStreetMap street network data within the urban extents from deblurred and corrected DMSP-OLS NTL RC 1996 image
	Connectivity	Intersection_density_1996	Node density of the set of nodes with more than one street emanating from them (Boeing, 2017)	OpenStreetMap street network data within the urban extents from deblurred and corrected DMSP-OLS NTL RC 1996 image
	Connectivity	Street_density_1996	Sum of all edges in the undirected representation of the street-network divided by the area of the urban extent	OpenStreetMap street network data within the urban extents from deblurred and corrected DMSP-OLS NTL RC 1996 image
III. Land use (*L*)	Sprawl	Sprawl_1996	Normalized difference between the share of pixels with population density below the regional average density and the share of pixels with population density above the regional average density (Fallah et al., 2011)	Population count at pixel level from GHS (GHS_POP_GPW41990_G LOBE_R2015A_54009_250_v1_0 at 250 meters of spatial resolution) within the urban extents from deblurred and corrected DMSP-OLS NTL RC 1996 data.
	Fullness	Fullness_1996	Fraction of built-up within the urban extent (Pesaresi et al., 2016)	Urban extents from deblurred and corrected DMSP-OLS NTL RC 1996 image; Built-up raster layer from GHS (GHS_BUILT_LDS1990_GLOBE_R2016A_54009_250_v1_0 at 250 meters of spatial resolution)

### Potential shape of urban extents

**Figure 5.**
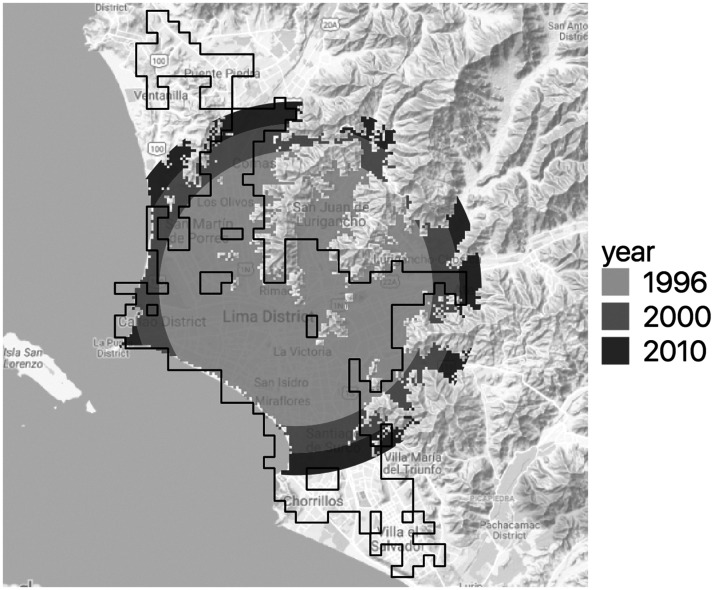
Lima 1996, extracted urban extent outline (black line) and potential urban extent areas for 1996, 2000, and 2010. Base map: ^©^Google.

As stated in the “Model” section, we follow Harari (2016) to produce a panel of city-year potential urban extents as instrumental variables. We use the digital elevation model from the NASA Shuttle Radar Topographic Mission (SRTM) version 4, with a resolution of 90 m (Jarvis et al., 2008), to calculate slopes and the Global MODIS Raster Water Mask (Carroll et al., 2009) to account for the presence of water bodies. In this study, we define steep terrains as those with a slope above 20%. This threshold is 5% steeper than the 15% suggested as the threshold for urban development in architectural development guidelines for developed countries (Saiz, 2010). This decision is because in LAC cities, urban development guidelines have been more tolerant, and cities have grown in areas with considerably steeper slopes. Figure 5 shows an example of an actual urban extent and its potential shapes on the three dates.

### Control variables

To isolate the predictive power of the variables describing urban form and to reduce omitted-variables bias, we include in the model a number of control variables, including population density, locational variables, and natural amenities, as well as country and year fixed effects. Regarding the fixed effects, we have 31 coefficients associated with country fixed effects and two coefficients associated with year fixed effects. As a common practice in the literature, those coefficients are not reported in the tables but the estimates are available upon author’s request (we indicate with a “Y” when these effects were included in the regression). Table 2 presents the control variables, descriptions, and data sources.

**Table 2.** Control variables (Vector *X*).

Dimension	Variable	Description	Data source for calculation
I. Urban population	Popden_1990	Population density within the urban extent	Population count at pixel level from GHS (GHS_POP_ GPW41990_GLOBE_R2015A_54009_250_v1_0 at 250 meters of spatial resolution) within the urban extents from deblurred and corrected DMSP-OLS NTL RC 1996 data (Freire and Pesaresi, 2015; Pesaresi et al., 2016)
II. Location	Dist_Border_kms_1996	Distance to the nearest international border	Country borders from the World Bank LAC Spatial Database and urban extents from deblurred and corrected DMSP-OLS NTL RC 1996 data
III. Natural Amenities	Temp_2010	Average annual temperature (C)	WorldClim, Bioclimatic variables, BIO1: annual mean temperature within the urban extents from deblurred and corrected DMSP-OLS NTL RC 2010 data (Hijmans et al., 2005)
	Precip_2010	Average annual precipitation (mm)	WorldClim, Bioclimatic variables, BIO12: annual precipitation within the urban extents from deblurred and corrected DMSP-OLS NTL RC 2010 data (Hijmans et al., 2005)
	Coast_2010	Dummy for location at the coast	MODIS Water Mask (Carroll et al., 2009) and urban extents from deblurred and corrected DMSP-OLS NTL RC 2010 data

## Empirical results

### Strategy 1: Lagged model

Supplemental Tables 1 and 2 provide basic descriptive statistics for the variables. The high correlation between *roundness* and *smoothness*, 0.74, precludes the inclusion of both variables in the same regression. The same situation occurs with *Intersection*_*density* and *Street*_*density* and *fullness* and *sprawl*, with correlations of 0.95 and −0.71, respectively. Table 3 presents the alternative specifications of the lagged model. The estimations use OLS and assume intragroup correlation; i.e. the residuals are correlated within the 32 countries in the sample but uncorrelated between them.

**Table 3.** Estimates of the relationship between urban form and productivity (OLS).

Variables	(1)	(2)	(3)	(4)	(5)	(6)	(7)	(8)
Roundness_1996	0.413***		0.389***		0.386***		0.351***	
	(0.0644)		(0.0638)		(0.0616)		(0.0609)	
Smoothness_1996		0.375*		0.351*		0.375*		0.339*
		(0.2001)		(0.1846)		(0.2025)		(0.1889)
Circuity_avg_1996	0.217	0.204	0.191	0.171	0.364	0.375	0.366	0.367
	(0.9388)	(0.8993)	(0.9112)	(0.8755)	(0.9687)	(0.9282)	(0.9432)	(0.9089)
Intersection_density_1996	0.488***	0.500***	0.495***	0.505***				
	(0.0870)	(0.0871)	(0.0967)	(0.0958)				
Street_density_1996					0.411***	0.423***	0.420***	0.430***
					(0.0688)	(0.0688)	(0.0788)	(0.0782)
Sprawl_1996	−0.083	−0.115			0.035	0.005		
	(0.1357)	(0.1605)			(0.1459)	(0.1757)		
Fullness_1996			0.484*	0.445			0.493*	0.449
			(0.2755)	(0.2917)			(0.2866)	(0.3061)
Fullness_1996^2^			−0.393*	−0.339			−0.468*	−0.409
			(0.2317)	(0.2331)			(0.2375)	(0.2446)
Constant	5.450***	5.542***	5.502***	5.266***	4.931***	4.936***	4.936***	4.924***
	(0.9338)	(0.8261)	(0.9461)	(0.7149)	(0.9369)	(0.8044)	(0.9210)	(0.7506)
Country dummies	Y	Y	Y	Y	Y	Y	Y	Y
Observations	919	919	919	919	919	919	919	919
R-squared	0.293	0.293	0.294	0.294	0.299	0.299	0.300	0.301

Robust standard errors clustered at the country level in parentheses. ***p < 0.01, **p < 0.05, *p < 0.1.

Controls: All models include *Popden_1990* and *Popden_1990^2^* and geographical characteristics as measured of natural amenities—namely, distance in km to international border, temperature, precipitation, and coast indicator.

*Intersection_density*_1996 has been divided by 100, *Street_density_1996* has been divided by 10,000, and the control *Dist_border_kms* has been divided by 1,000.

The dependent variable is the logarithm of the density of radiance within the urban extent, ln(dr_ntl_2010).

Concerning the shape of the urban extent, the results show positive and highly significant coefficients for *roundness* and positive but less significant coefficients for *smoothness* across all specifications. These results provide support for hypothesis H1, according to which, all else unchanged, a more circular urban extent and a smooth perimeter are correlated with higher productivity levels. Up to this point, we found empirical evidence of the association between the first dimension of urban form (shape) and productivity.

Regarding the urban structure within the city (our second dimension of urban form), we found no evidence in favor of hypothesis H2. The nonsignificance of *circuity* across all specifications implies that, after controlling for other measures of urban form, the level of productivity of the city does not correlate with the presence of a reticular or an organic urban structure. Conversely, the results provide evidence that supports hypothesis H3. The level of connectivity, measured as either *Street_density* or *Intersection_density*, appears positive and highly significant in all specifications, suggesting that, other things held constant, dense street networks are associated with higher productivity. Thus, the urban structure variables show us that, in terms of productivity, what matters is the high intra-urban connectivity, regardless of whether this is achieved through a reticular or organic urban structure.

Finally, the results for the analysis of our third dimension (land use) vary across specifications. Contrary to the results presented by Fallah et al. (2011), the *sprawl* variable is not significant across all specifications, which suggests that, after controlling for the shape and the urban structure of the city, there is no evidence of correlation between the distribution of population density within cities and their productivity levels (i.e. we found no evidence supporting hypothesis H4). In those models in which we use the variable *roundness* to control for the shape of the urban extents, we obtain significant and expected signs for the variables *fullness* and *fullness^2^* (see estimations 3 and 7 in Table 3). The coefficient of *fullness* is positive and significant, showing how less interrupted urban layout (i.e. low urban sprawl) is correlated with higher productivity levels. Yet, the coefficient of *fullness*^2^ is negative and statistically significant, suggesting that an excessive fullness may reflect a lack of public space and/or a lack of planning, is correlated with the appearance diseconomies of agglomeration. These results support the hypothesis H5. However, the significance of *fullness* and *fullness*^2^ disappear when we use the variable *smoothness* to control for the shape of the urban extents (see estimations 4 and 8 in Table 3). We discard the presence of multicollinearity after a careful inspection of the variance inflation factors (VIFs) for the independent variables in estimates 4 and 8 in Table 3.

According to the coefficient estimates reported in Table 3, 10% change in the *roundness* (*smoothness*) index would be associated with productivity levels that are about 3.9% (3.6%) higher. Regarding the urban structure, the coefficient estimates indicate that 100-units (10,000-units) change in the *intersection_density* (*street_density*) would be associated with productivity levels that are about 50% (42%) higher. Although these estimates seem high, it is important to note changes in any of these variables are not easy to achieve. For instance, on average, *roundness*, *smoothness*, *intersection_density*, and *street_density* in LAC cities decreased 0.01, 0.03, 13.93, and 1,892.03 units respectively between 1996 and 2010.

### Strategy 2: Instrumental variables

The implementation of instrumental variables techniques was carried out using the two-stage least squares estimator (2SLS), allowing intragroup correlation at the country level. In Table 4, we report the 2SLS estimations, which use the log of normalization of potential urban form (*roundness* and *smoothness*) as instruments for the actual urban form. We present in Supplemental Table 4 the estimates of the first stage. Supplemental Table 3 shows the descriptive statistics of the variables.

**Table 4.** Estimates of the relationship between urban form and ldr by OLS and 2SLS. Dependent variable: ln(dr_ntl)

	OLS (1)	2SLS (IV: Norm. potential roundness) (2)	OLS (3)	2SLS (IV: Norm. potential smoothness) (4)
Roundness	0.672***	0.958**		
	(0.1305)	(0.4298)		
Smoothness			0.366***	−0.370
			(0.1198)	(0.3930)
Country dummies	Y	Y	Y	Y
Year dummies	Y	Y	Y	Y
N	2757	2757	2757	2757
R-squared	0.274	0.142	0.262	0.090
**Instrument relevance**				
**1. First-stage statistics**				
*F*-stat^a^		62.37		29.22
*F*-stat *P*-val		0.000		0.000
**2. Underidentification test**				
Kleibergen–Paap rk LM stat^b^		4.481		5.601
Chi-sq *P*-val		0.0343		0.0179
**3. Weak identification test**				
Kleibergen–Paap rk Wald *F*-stat^c^		62.368		29.218

Robust standard errors are clustered at the country level in parentheses. ***p < 0.01, **p < 0.05, *p < 0.1.

All models include geographical characteristics as measured of natural amenities—namely, temperature and coast indicator.

^a^*F*-test of excluded instruments (*roundness* or *smoothness*) in the first stage model. *F*-stat above 10 indicates that the instrument has considerable explanatory power.

^b^The Kleibergen–Paap rank LM test of underidentification tests whether the excluded instruments are correlated with the endogenous regressor.

^c^The Kleibergen–Paap rank Wald test of weak identification tests the significance of the excluded instruments in the structural equation. The critical values for this test are from Stock and Yogo (2005). The values of this test are higher than the Stock and Yogo (2005) critical values, suggesting that the instruments are not weak.

We began by discussing the instruments diagnostic test reported at the bottom of Table 4. Regarding the relevance of the instruments, the first stage of regression results shows that the instruments for actual urban form have considerable explanatory power. The explanatory power is tested using the *F*-tests; we find values above 10, indicating that these instruments are strongly related to actual urban form. In fact, the results from estimating the first stage, reported in Supplemental Table 4, show that the potential urban form is a highly significant and positive predictor of the actual urban form for *roundness* as well as *smoothness*. To further inspect the relevance of the instruments, we carried out a Kleibergen–Paap test of underidentification (Kleibergen and Paap, 2006), which tests whether the model is identified, with identification requiring that the excluded instruments are correlated with the endogenous regressor. The values of this test for the two models indicate rejection of the null hypothesis of underidentification at a 5% level of significance, suggesting that the instruments are relevant. We also performed a weak instrument test to assess whether the instruments are only weakly correlated with the endogenous regressors. Since we allow intragroup correlation, the relevant statistic in this case is the Kleibergen and Paap (2006) rank Wald *F*-statistic. The results reveal that the statistic values are higher than the Stock and Yogo (2005) critical values, rejecting the null hypothesis of weak instruments.

We now turn to consider the estimates of the impact of urban form on productivity. For the case of *roundness*, we note that both the OLS and 2SLS estimates reveal that a higher level of urban compactness is associated with higher levels of productivity. The coefficient on *roundness* is positive and highly significant at the 5% level, and the 2SLS coefficient is higher than what was found with the OLS estimate. Regarding *smoothness* estimates, the results show that while the OLS estimate is positive and significant in statistical terms, the 2SLS coefficient is not significant.

Because of the lack of information, the panel in these IV specifications uses irregular time intervals (1996, 2000, and 2010), which makes the size of the coefficients hard to interpret. Therefore, we suggest that these estimates should be treated as indicative.

## Conclusions

This paper uses a more integral way of measuring urban form and its relationship with economic performance. Instead of using only population density as a proxy for urban form, we use seven variables that cover the three dimensions of urban form: shape of the urban extent, structure of the urban texture, and land use patterns. For the empirical evidence, we use two strategies for dealing with the endogeneity between urban form and productivity: first, using lagged explanatory variables, as in Fallah et al. (2011) and, second, using instrumental variables, as in Harari (2016).

Based on our findings from 919 cities in the LAC region, we can conclude that both the shape of the urban extent and the structure of the urban texture have an impact on city productivity. Cities with rounded/compact and smooth perimeters and dense street networks meet important conditions for being highly productive. These results imply that urban planning tools, such as land use and transportation planning, infrastructure investment, and other zoning regulations, not only determine the form in which cities grow but can also affect their productivity levels through their impact on the three dimensions of urban form: shape, structure, and land use.

An important consequence of decomposing the concept of urban form into three dimensions is that it points at different instruments policy makers can use to increase productivity in their cities. Our work indicates that a noncompact city can reach high levels of productivity by guaranteeing a high level of inner-city connectedness; alternatively, it may happen that a compact but poorly connected city can show low levels of productivity. In summary, our empirical evidence shows that each city can find its way towards higher productivity levels by analyzing the status of each dimension of its urban form and implementing corresponding strategies to improve its current conditions.

Finally, in this work, we show the benefits of using open data that are available on a global scale and open source tools. Further research can use the same data sources and methodological strategies to provide additional and comparable evidence for other regions in the world.

## Supplemental Material

sj-pdf-1-epb-10.1177_2399808321999309 - Supplemental material for Urban form and productivity: What shapes are Latin-American cities? Supplemental material, sj-pdf-1-epb-10.1177_2399808321999309 for Urban form and productivity: What shapes are Latin-American cities? by Juan C Duque, Nancy Lozano-Gracia, Jorge E Patino and Paula Restrepo in EPB: Urban Analytics and City Science
