# Supplementary material for: Urban form and productivity: What shapes are Latin-American cities?
Source: Environ Plan B Urban Anal City Sci. 2021 Mar 8;49(1):131–50. doi: 10.1177/2399808321999309 (PMC13021087; doi:10.1177/2399808321999309)
Supplement: sj-pdf-1-epb-10.1177_2399808321999309 - Supplemental material for Urban form and productivity: What shapes are Latin-American cities? [file sj-pdf-1-epb-10.1177_2399808321999309.pdf]

Supplementary Table 1. Descriptive statistics of the data used in (2).

Variable	p25	Median	p75	Mean	Std. Dev.	Min	Max
dr_ntl_2010	360.067	514.331	733.474	587.184	318.228	170.060	3,759.463
Roundness_1996	0.725	0.828	0.879	0.787	0.121	0.266	0.947
Smoothness_1996	0.620	0.720	0.767	0.674	0.140	0.090	0.888
Circuity_avg_1996	1.019	1.028	1.046	1.037	0.034	1.002	1.419
Intersection_den_1996	46.956	64.092	84.136	65.867	28.881	0.333	184.972
Street_den_1996	7,988.395	10,452.410	12,988.890	10,474.380	3,791.514	135.913	20,669.240
Sprawl_1996	0.478	0.595	0.721	0.598	0.177	0.108	1.000
Fullness_1996	0.494	0.630	0.763	0.618	0.192	0.014	0.996
Popden_1990	0.106	0.163	0.236	0.188	0.123	0.003	0.872
Dist. (Km) inter. border	152.164	413.717	765.123	555.744	542.225	0.000	2,461.340
Temperature	18.954	22.133	25.476	21.742	4.331	5.486	28.892
Precipitation	838.185	1,244.574	1,532.889	1,239.906	667.526	0.519	7,250.133
Coast	n/a	n/a	n/a	n/a	n/a	0	1

Supplementary Table 2. Correlation matrix between urban form variables.

Variables	(1)	(2)	(3)	(4)	(5)	(6)	(7)
(1) Round._1996	1.000						
(2) Smooth._1996	0.745*	1.000					
(3) Circ._avg_1996	-0.199*	-0.117*	1.000				
(4) Int._dens_1996	0.143*	-0.031	-0.390*	1.000			
(5) St._dens_1996	0.153*	-0.047	-0.435*	0.947*	1.000		
(6) Sprawl_1996	0.042	0.222*	0.203*	-0.545*	-0.594*	1.000	
(7) Fullness_1996	-0.157*	-0.310*	-0.059	0.343*	0.436*	-0.712*	1.000

* significance at the .05 level

Supplementary Table 3. Descriptive statistics of the data used in (3) and (4).

Variable	p25	Median	p75	Mean	Std. Dev.	Min	Max
dr_ntl	329.377	465.960	676.922	545.439	310.412	138.902	3,759.463
Norm. actual roundness	0.720	0.823	0.879	0.786	0.121	0.266	0.952
Norm. actual smoothness	0.596	0.712	0.760	0.660	0.143	0.090	0.888
Norm. potential roundness	0.888	0.977	1.000	0.927	0.104	0.245	1.000
Norm. potential smoothness	0.505	0.796	1.000	0.726	0.272	0.095	1.000
Temperature	18.979	22.147	25.475	21.756	4.323	5.486	28.897
Coast	0.000	0.000	0.000	0.173	0.379	0.000	1.000

Supplementary Table 4. Estimates of the first stage of instrumental variables regressions

	OLS<br>Dependent var:<br>Norm. actual roundness<br>(1)	OLS<br>Dependent var:<br>Norm. actual smoothness<br>(2)
Norm. potential roundness	0.263***	
	(0.0333)	
Norm. potential smoothness		0.118***
		(0.0218)
Country dummies	Y	Y
Year dummies	Y	Y
N	2,757	2,757

Note: Robust standard errors are clustered at the country level in parentheses.
*** p<0.01, ** p<0.05, * p<0.1
All models include geographical characteristics as measured of natural amenities—namely, temperature and coast indicator.
